# Competencies of nurses to participate in safe medication management practices for biologics: A scoping review

**DOI:** 10.1371/journal.pone.0317750

**Published:** 2025-01-27

**Authors:** Wansheng Li, Li Li, Linbo Li, Cardenas Xiaodong, Mudiao Chen, Hongye Liu, Peirao Li

**Affiliations:** 1 College of Nursing, Shanxi Medical University, Taiyuan, China; 2 Nursing Department, The First Hospital of Shanxi Medical University, Taiyuan, China; 3 Department of Psychiatry, The First Hospital of Shanxi Medical University, Taiyuan, China; 4 Japanese Red Cross Toyota College of Nursing, Toyota, Japan; 5 Nursing Department, Dermatological Hospital of Southern Medical University, Guangzhou, China; 6 Department of Dermatology, The First Hospital of Shanxi Medical University, Taiyuan, China; 7 Nursing Department, Shanxi Mental Health Centre, Taiyuan, China; Birjand University of Medical Sciences, IRAN, ISLAMIC REPUBLIC OF

## Abstract

**Aim:**

To review the existing literature relating to nurse competence in safe medication management practices for biologics, identify evidence, and develop a competency framework to clarify the role of nurses in these practices.

**Background:**

With the widespread use of biological agents in disease treatment, ensuring the safe and economical use of high-cost medicines is particularly important. Even though nurses are essential in patient care, detailed knowledge regarding their competence and role in the safe administration of biologics is lacking.

**Design and methods:**

A scoping review was performed following the methodology of Arksey and O’Malley and the PRISMA ScR guidelines. Electronic databases, including PubMed, CINAHL, Embase, Scopus, and Web of Science, were searched using accepted keywords, and relevant articles were identified using inclusion and exclusion criteria.

**Results:**

A total of 3,422 studies were retrieved, 24 of which were eligible for inclusion. The required competencies for nurses were summarized into six areas: clinical specialized knowledge, critical thinking and problem-solving skills, safe medication skills, health education skills, communication and coordination skills, and technological literacy.

**Conclusion:**

We provide insights into the competencies of nurses involved in the safe medication management of biologics. These competencies can be used to assess the actual competency level of nurses and facilitate the maximization of biological treatment goals and outcomes. This plays a vital role in optimizing the use of healthcare resources and demonstrating outcomes.

## Introduction

Biologics have a wide range of applications in various diseases, including autoimmune, oncological, infectious, and genetic, owing to their targeted efficacy, rapid onset of action, and tolerability [1,2]. With the widespread use of biologics in clinical practice, the development of adverse events, such as allergies and other pharmacologically related side effects, is a major barrier to continuing treatment despite their significant therapeutic benefit [3]. To parovide effective and cost-efficient treatment with biologics, it is imperative to optimally conduct their safe management. This necessitates careful coordination and supervision of various factors by professionals, including screening and identifying potential contraindications to treatment, monitoring and managing drug reactions and side effects, safety assurance, and patient education [4-5]. Nurses are one of the largest groups providing frontline care and play an important role in safely administering biologics [6]. They also act as executors of medical advice and directly participate in the daily care of patients [7].

The World Health Organization emphasizes the pivotal role of nurses in primary healthcare as educators, coordinators, and providers of home-based care [8]. Their professional training makes them indispensable for illness prevention, diagnosis, treatment, management, and rehabilitation to ensure safe and efficient patient care [8]. The American Nurses Association (ANA) has devised a competency framework for nursing professionals based on U.S. laws, regulations, and nursing policies pertaining to knowledge, skills, ability, and judgment [9]. Based on this framework, managing the safe administration of biologics requires nurses to possess a comprehensive range of competencies in delivering nursing care. These include preparing and safely administering biological agents, risk assessment and prevention, emergency management, health education, and psychological support [10,11].

In addition, to alleviate the financial burden on patients, some countries are actively exploring and developing biosimilars that offer the same quality, safety, and efficacy as the original biologics but at a lower cost. Since their approval by the European Medicines Agency in 2004 [12], biosimilars have made biological treatments more accessible and improved patient care. Even without direct prescribing authority, nurses are essential in explaining to patients the rationale behind switching to biosimilars, as highlighted by the Royal College of Nursing [13]. This underscores the expanding responsibilities of nurses to ensure the safe and effective administration of biologics [14,15].

The literature indicates that nurse-led biologic practices enhance patient monitoring, therapy appropriateness, and quality of life while reducing medication costs [16]. A systematic review of 13 articles showed that nurses received positive feedback on disease control and achieved higher satisfaction in patient self-care and treatment adherence compared with physicians [17]. As healthcare shifts toward population health, nursing practices must adapt to evolving patient and system needs, including understanding patient preferences, needs, and values and intervening at the population level. Despite challenges in patient-centered care, health promotion, and interdisciplinary collaboration, a comprehensive nursing competency framework has yet to be established.

After our initial search and review, we identified several studies that described nurses’ competencies in administering biologics [12,18,19]. However, we found that most of these studies were limited to a single clinical skill or specific competency and did not take a holistic, systematic, and comprehensive approach to examining a wider range of nurses’ competencies [20]. Scoping reviews, an innovative method for synthesizing research, provide a comprehensive literature overview and are particularly useful for complex or under-reviewed topics [21]. Therefore, we conducted a scoping review to develop a nursing competency framework. This framework was constructed based on an analysis of the extant evidence pertaining to nurses’ competencies in the safe management of biologics. It further elucidates the nursing role and scope of practice and informs and inspires the education and training of clinical nurses.

## Methods

The methodological approach of this review was based on Arksey and O’Malley’s (2005) framework for scoping reviews [22], which involves (i) identifying the research questions, (ii) searching for relevant studies, (iii) selecting studies, (iv) charting the data, and (v) collating, summarizing, and reporting the results. The Preferred Reporting Items for Systematic Reviews and Meta-Analyses Extension for Scoping Reviews (PRISMA-ScR) guidelines were followed to ensure satisfactory reporting [23] (S1 Table).

### Identifying the research questions

We used the population, concept, and context (PCC) framework recommended by the Joanna Briggs Institute for scoping reviews [24]. Table 1 presents the PCC framework that defines our research questions and search strategies. The main research question was, “What competencies are required for nurses to participate in safe medication management practices for biologics?”

**Table 1:** Inclusion and exclusion criteria Include.

	Include	Exclude
**Population**	General nurses Registered Nurses Licenced practical nurses Advanced practice nurses (Nurse Specialist and Nurse Practitioner) Nurse managers	Pre-registration nursing students Patients, family members Carers
**Concept**	Nursing Practice activities (competencies, role, knowledge, skills, capacity, attitude and abilities)	Publications that focused on management and education
**Context**	Family practices and home visits(including adult practices, internal medicine practices, community health centres) Hospital settings (such as acute hospital, community hospital)	Nursing homes, Long-term care facilities
**Types of sources**	Experimental and quasi-experimental study designs Analytical and descriptive observational studies Qualitative studies Dissertations, theses, and websites of national nurse Practitioners’ organisations Literature review Editorial paper	Study protocols Summaries/comments/discussions

### Searching for relevant studies

We applied a three-step search strategy for this review,which was developed through collaboration between the research team and subject experts. The search strategy was guided by the PCC framework, and an initial pilot search was conducted by two researchers in PubMed and CINAHL. During this pilot search, we identified articles and analyzed the words in the title and abstract, as well as the index terms used to describe the article. The keywords were subsequently identified by employing a combination of Boolean operators. The search strategy was derived from two main concepts: (i) nursing competencies, role, knowledge, skills, capacity, attitude, and abilities and (ii) biologics, biological agents, biological agents, or biosimilars. During the second search, we considered all the keywords and index terms identified across the databases. In the third search, we examined the reference lists of all included reports and articles to identify additional studies. The complete search strategy is provided in S2 Table. We systematically searched PubMed, CINAHL, Embase, Scopus, and Web of Science for articles published from their inception to May 2024. The articles were exported and managed using NoteExpress 3.8 and Microsoft Excel, and PowerPoint was utilized to create the figures.

### Selecting studies

#### Inclusion and exclusion criteria.

The development of this section also adhered to the PCC model [24]. The population (P) refers to nurses involved in the safe medication management of biologics, including general and registered nurses. Pre-registration nursing students and others were excluded. The concept (C) focuses on nurses’ competencies, roles, and knowledge. Context (C) includes family practices, home visits, and hospital settings. Details of the specific inclusion and exclusion criteria are provided in Table 1.

#### The screening process.

Each included study was screened by at least two research team members (LLB and LHY). Full-text screening and discussion of divergences were performed. A third reviewer was consulted to resolve any disagreements.According to the guidelines of Arksey and O’Malley, we did not perform risk of bias or quality assessments for the included studies [22].

#### Charting the data.

Data from all included studies were extracted and categorized into subgroups based on the following headings: author, year of publication, country, study objective, study design, nurses’ competencies in Nursing Scope and Standards of Practice (NSSP) [9], and competencies relevant to safe medication management practices (S3 Table). One reviewer extracted the data, and another cross-checked the data extraction. The first author reviewed all the included studies to ensure that no information was missing.

#### Collating, summarizing and reporting results.

**Fig 1:**
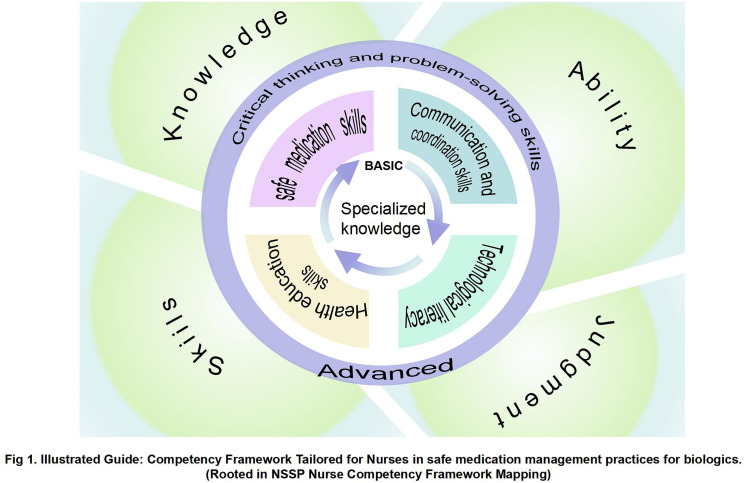
lllustrated Guide: Competency Framework Tailored for Nurses in safe medication management practices for biologics.

The extracted data are summarized and reported in the tables and figures. Nursing competencies are mapped per the recommendations in the NSSP 4th Edition. The NSSP comprises four domains: (A) knowledge, (B) skills, (C) ability, and (D) judgment [9]. Additional competencies are summarized separately in S3 Table. This is a suitable framework for developing a comprehensive understanding of the competencies identified in the included studies. Finally, we created a plot linking the four domains of the NSSP and other competencies (Fig 1).

## Results

### Study description

**Fig 2:**
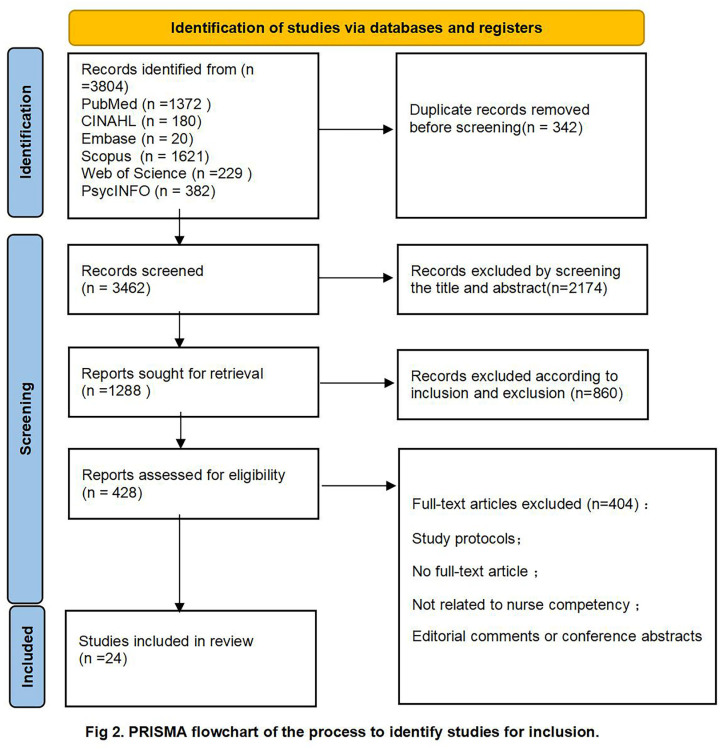
PRlSMA flowchart of the process to identify studies for inclusion.

A total of 3,422 records were generated across 5 databases; 340 duplicate reports were removed, and 2,070 results were screened by title and abstract. Finally, 24 studies were included in this review. The PRISMA flowchart describes the study selection and inclusion processes (Fig 2). The 24 articles were conducted primarily in 11 countries: Denmark, Italy, the United Kingdom, Germany, Sweden, Israel, Switzerland, the United States, France, Canada, and Spain. These articles were published between 2010 and 2023, with a gradual increase in the number of articles published after 2016. The study designs are shown in S3 Table.

### Framework for nurses’ practical competence in managing the safe administration of biologics

#### Clinical specialized knowledge.

Clinical specialized knowledge is the theoretical basis for all nursing practice activities. We defined clinical specialized knowledge as multidisciplinary theoretical knowledge and skills that nurses need to participate in biologics therapy rather than focusing on one disease area. Coordination and management in safe medication management of biologics require nurses to have a deeper and broader knowledge base to provide support, education, management, assessment, and monitoring of patients [25,26]. Sixteen studies indicated that the biologics-related knowledge required by nurses included, but was not limited to, the following: knowledge related to the disease, biologics administration and use, pharmacology, nursing, and therapeutic procedures, management of adverse reactions or events, and medication precautions for special conditions or populationss.

#### Critical thinking and problem-solving skills.

Critical thinking is a prerequisite for developing all other competencies. This mode of thinking applies to every aspect of a competency. Nurse duties are not limited to administering biologics or dealing with allergies and other adverse reactions that may occur on the day of infusion or a few days later. Nurses can use their knowledge and judgment to proactively set goals and provide anticipatory patient care before an adverse event occurs. The report indicates that trained nurses can independently manage the initial stages of an acute infusion reaction, thereby allowing the patient to receive optimal resuscitation care [27]. In complex clinical scenarios where unexpected situations are inevitable, nurses are expected to assess, analyze, and make judgments promptly and accurately to make evidence-based decisions. In addition, they are expected to engage in self-regulated judgment and reflection after resolving clinical care issues, facilitating the development of self-directed learning abilities and thereby enhancing their practices.

#### Safe medication skills.

The medication management process is susceptible to errors because of its intricate nature and comprises five steps: prescribing, checking, preparation/dispensing, administration, and monitoring. One-third of medication errors in hospitals occur during the medication configuration and administration phases, which are dominated by nursing activities. Several studies have asserted that nurses should be proficient in the safe administration of medications to ensure their efficacy and safety. This encompasses a range of topics, including the administration and storage of medications, assessment and risk screening of patients before beginning medication therapy, monitoring of medication side effects and adverse reactions, and timely reporting of adverse events.

#### Health education skills.

Twenty-two studies demonstrated that nurses are well-positioned to provide education on biologic agent therapy, thereby confirming patients’ implementation of this information in their daily lives. Prior to administering medication, nursing staff are responsible for disseminating information to the patients on the potential benefits of biologics, expected efficacy, risk of adverse effects, length of treatment, and economic costs. Moreover, nurses must educate patients during medication administration on monitoring for adverse reactions and basic management, as well as on the importance of regular follow-ups and avoiding live vaccines. To optimize efficacy, patient education on self-management following medication administration should encompass guidance on healthy living (diet, sleep, and psychological support) and weight management. Furthermore, nurses should provide instructions on self-injection techniques while ensuring that patients are fully informed about the dosage, frequency, and storage requirements of the medications. To enhance the efficacy of patient education, assessment tools that consider the individual educational requirements of patients, such as the Educational Needs Assessment Tool, are recommended.

#### Communication and coordination skills.

The communication and coordination skills of nurses are of great importance in biologics therapy. They are necessary for healthcare professionals to continuously communicate with patients to address potential problems [18]. These may include dissatisfaction with the treatment outcome owing to a lack of information in different patient populations. Building trust with patients throughout the treatment process is essential. This can be achieved by conveying accurate information, collaborating in problem-solving, and increasing satisfaction. The study comprises the following main themes: multidisciplinary collaboration, facilitating shared decision-making, and building a strong, trusting, and therapeutic relationship versus providing personalized, humanistic care and support. The study indicates that nurses are pivotal in facilitating communication between patients and doctors during treatment [28]. They also disseminate crucial information to other healthcare professionals, contributing to the accuracy of diagnoses and formulation of personalized treatment plans. This ultimately enhances the quality of care and patient satisfaction while fostering a harmonious doctor-patient relationship.

#### Technological literacy.

In this scoping review, we also emphasize the significance of nurses’ utilization of mobile information and digital health technologies in the context of biological nursing practice. Nurse-led telecare provides real-time monitoring, health counseling, and self-injection guidance to patients through remote monitoring, training, coaching, and counseling, as well as remote home care using telemedicine technologies to implement nursing care for patients and guide nursing practice. Additionally, nurse-led telemedicine, encompassing patient counseling hotlines and virtual clinics, facilitates effective communication with patients and their self-management and influences healthcare utilization and cost savings [7,17,29]. Furthermore, this study indicated that nurses should possess the requisite skills to utilize computerized management systems and apply nursing informatics tools to facilitate clinical nursing practice [16].

## Discussion

In this scoping review, we summarize nurses’ competencies related to safe medication management of biological therapy. The research team examined and selected 24 articles and identified 6 competencies that were complex and challenging to categorize because most of the elements were interrelated. The theoretical foundation of this study was derived from the framework of nursing practice competencies developed by the ANA [9]. This framework is a universal guideline that aims to comprehensively cover and enhance nurses’ professional skills and practices. On this basis, we conducted in-depth analyses and expanded, refined, and strengthened the framework in biologics medication management. Six detailed and specific sub-frameworks were refined and constructed to provide a set of scientific, systematic, and operable competency standards and an evaluation system for biologics nursing practice.

Within this study’s framework, clinical expertise was the most essential competency for nurses to manage biologics in clinical practice. It encompasses the comprehension and mastery of professional knowledge concerning the mechanism of drug action, methods of administration, points of observation, the distinctive characteristics of each class of drug, and the adverse reactions that may be induced [30]. Some studies have indicated that nurses’ lack of knowledge and understanding of biologics may affect patients’ trust and willingness to use them [31]. Concurrently, nurses assume a pivotal role in the transition between biologics and biosimilars. As the primary conduit of patient data, nurses must thoroughly understand the policies and regulations prevailing in their country, region, or hospital. Moreover, they must apply these policies as a framework during the drug-switching process to provide scientifically based standardized safety information, medication reimbursement [19,32], and health insurance policies for patients undergoing treatment and for special populations [33,34]. To further this expertise, continuing nursing education is essential to enhance their understanding of biologics and equip them with the knowledge needed for potential prescriptive authority in the future [35].

The essence of a nurse’s competence in implementing safe medication management is their overall internal motivation, knowledge, and skills to use critical thinking to address safety issues related to administering biologics in real-world care settings. This area of competence directly impacts the quality of care and patient safety [36,37]. In clinical practice, nurses must follow strict standard operating procedures for infusion, have the necessary skills to administer infusions, and be able to analyze the relationship between patient symptoms and available evidence to identify potential causes of side effects or adverse reactions [38]. In addition, nurses must recognize signs of abnormalities promptly, develop appropriate care plans, and document the safety precautions that have been addressed [7,39]. Improving nurses’ problem-solving skills is essential for enhancing their self-efficacy, coping skills, and application of nursing procedures [40]. However, deficiencies in independent problem-solving and evidence-based care among nurses responsible for biologics therapy should be addressed by providing systematic theoretical and practical training to enhance theory-based coping skills in clinical practice [41].

Nurses are the primary implementers of various clinical drug treatments and are responsible for ensuring the safety of drug administration [10]. They are the primary source of nursing care and are responsible for providing quality care [42]. Biologics, as a new biologically active agent, present several unique risks, including denaturation and inactivation of the agent owing to contamination or inappropriate storage and transport conditions, as well as potential efficacy and safety issues related to unregulated clinical use [43]. Nurses are regarded as the foundation of safe medication practices. Before administering biologics, a comprehensive evaluation of a patient’s medical history and meticulous screening for tuberculosis, viral hepatitis B and C, herpes zoster, HIV, and opportunistic infections should be conducted. Biologics are contraindicated in patients with severe active infections and severely compromised immunity [38]. The elevated burden of treatment and the prevalence of post-medication adverse effects can be reduced by nurse-led interventions, including early identification and reporting of adverse events, effective monitoring of high-risk medications and patients’ vital signs, and the use of adjuncts to physicians’ use of antihistamines and hormones prior to treatment with biologics [44]. Notably, nurses do not provide direct prescription services in the context of biologics nursing practice. This may be attributable to financial constraints or other factors. However, nurses exert some influence on prescription decisions when handling adverse reactions [45].

Nurses must possess the ability to evaluate current practices and use relevant knowledge and guidelines to improve their practice, including evidence-based practice [19]. Therefore, accurate health advice must be provided. Existing research focuses on patient-centered care involving basic treatment, care, and dissemination of educational and supportive resources. Nurses can enhance patients’ self-management abilities, self-efficacy, and overall well-being [46]. Weight fluctuations affect medication dosage, and unhealthy lifestyles reduce treatment efficacy [47]. Nurses should reinforce patient life management guidance through health education to enhance patients’ self-management abilities. For immune-mediated polygenic diseases, such as psoriasis, nurses can diagnose high-risk populations based on genomic information before treatment and provide genetic counseling during treatment [18,48]. It is incumbent upon nurses to ensure that patients, as consumers of healthcare and recipients of treatments, are provided with appropriate information about the cost of biologic treatments. This should include, but not be limited to, details of the pricing mechanism of the drug, possible out-of-pocket costs, and reimbursement coverage and conditions under different health insurance plans. By providing explanations and comparative analyses, nurses can assist patients in comprehending the treatment’s financial implications, thereby enabling them to make informed decisions. In special populations, such as pregnant and lactating women, infants, and children, it is of the utmost importance to provide comprehensive information regarding the risks, safety, and potential benefits of the medications in question. Furthermore, the decision to use biologics should be made after thoroughly weighing the advantages and disadvantages of the treatment in conjunction with the patient [30,49]. Providing mental health support in the context of health education greatly enhances patient confidence and compliance with treatment, relieves tension and anxiety, and avoids any emotional impact on the treatment process. Concurrently, nurses have employed a range of interventions, including positive thinking, cognitive behavioral therapy, and other non-pharmacological therapies, to facilitate the implementation of psychological counseling for patients [43]. Nurses are expanding the boundaries of conventional nursing practices. Given the ever-changing landscape of medical knowledge and clinical demands, nurses must engage in lifelong learning and professional development to remain relevant in their practices.

The existing research also highlights the need for nurses to acquire communication and coordination skills, which are crucial in shaping their future professional roles [50]. Several qualitative studies suggest that patients are more likely to communicate with nurses because they believe in their ability to develop deep emotional connections [16,51,52]. A survey conducted in France showed that patients who did not benefit from nurse counseling had lower self-management safety skills [53]. Nurses implemented a meticulous communication strategy during the care process for first-time and long-term patients treated with biologics. This strategy allowed for immediate feedback on drug efficacy and signs of drug resistance, which provided critical data to support physicians in accurately adjusting treatment dosages. This interaction has the dual benefit of enhancing the treatment plan’s personalization and effectiveness while promoting timely optimization of the treatment pathway. This highlights the active role of nurses in promoting dynamic adjustments of treatment plans. Nursing practice based on shared decision-making (SDM) emphasizes the role of the patient in the treatment process [19]. Nurses encourage patients to provide timely feedback on their health needs to actively participate in clinical decision-making with full consideration of their values and preferences. This involvement in managing their condition promotes SDM and ensures the continuity of care with biologics. Nurses should place the patient at the center of the decision-making process [38], focusing on the patient’s needs at any given time and considering factors such as family planning and breastfeeding before treatment, proximity to the hospital during treatment, employment or education issues, ability and willingness to self-inject, and consistency [25]. All information nurses provide should be personalized and discussed with the patient to promote SDM.

Competence in clinical practice should not be understood at the level of purely practical operations but should focus more on the need for nurses to demonstrate a more flexible understanding and responsiveness in handling different clinical situations. Nurses should collaborate and coordinate care with other professionals to ensure streamlined treatments. They often act as focal points in multidisciplinary collaborative processes and work with professionals from other disciplines. Multidisciplinary team virtual clinics proactively provide patients with diagnoses, treatments, and care approaches and reduce costs associated with biologics by improving de-escalation rates and reducing costs associated with biologics treatments [54]. These clinics also reduce the costs associated with biologics treatments by increasing step-down rates and decreasing outpatient visits by reducing the number of patient encounters [55]. Clarifying the roles, responsibilities, and scope of practice of multidisciplinary biologics clinics provides a methodological basis for subsequent multifaceted and ongoing evaluations of the effectiveness of multidisciplinary care implementation.

The ability of nurses to use information technology has emerged in the field of biologics therapy. The Electronic Biologics Management System digitizes nurse-led drug therapy monitoring by automatically collecting and continuously updating patient data at the point of care [16]. Nurses use a web-based self-management system to provide informational, educational, and psychological counseling to patients receiving biologics therapy, improving disease awareness and adherence. Nurse-led telecare technology has shown positive results during patients’ treatment with biologics and follow-up visits and was well received by patients during the COVID-19 pandemic [56]. Maintaining continuous communication with patients through information and communication technology video calls for long-term disease monitoring and timely recognition and management of infusion reactions, is important. This pathway is similar to but not a substitute for nurse-led multidisciplinary virtual biologics clinics, and telecare is a reliable option for improving the continuity and timeliness of care [29]. In addition, some patients are still concerned about the economics and practicality of telecare and whether it affects privacy [57]. Therefore, we will continue to be guided by the individual needs of patients. Moreover, we will thoroughly evaluate the impact of nurse-led telecare on patients treated with biologics, provide better training for healthcare professionals implementing telecare, expand the content of telecare, and improve the telecare service system in line with current trends under the premise of protecting personal privacy.

The primary objective of our competency framework was to deliver person-centered quality care, emphasizing the need for nurses to foster an environment conducive to quality care, address patients’ physical and psychological needs, and offer personalized and humanized support and care. This approach ensures respect for patients’ dignity, autonomy, and privacy during treatment, adheres to principles of justice and fairness, and promotes the equitable distribution of medical resources, aligning clinical nursing practice with humanitarian needs and societal development [58]. Using this framework, we conducted a comprehensive and structured review of the competencies essential for nurses’ participation in safe biologics management. By enhancing the professionalism of nursing teams with clear practice competencies, we aimed to identify more efficient working models that optimize the goals and outcomes of biologics therapy. This helps achieve optimal resource utilization and outcomes without substantially increasing healthcare staff. Future research should enhance nurses’ medication management competence through education and training and optimize biologics’ clinical use through interdisciplinary collaboration. The ultimate goal is to provide safer and more effective care for patients and continuously refine safe management practices, thereby advancing the quality and safety of patient care in the context of biologics therapy.

### Limitations

This study has some limitations that should be considered. First, only English-language articles were included, which might have introduced publication bias; however, we used multiple methods to search five databases to minimize the omission of articles. Second, content regarding nurses’ prescribing rights may be lacking in our study. Specific laws, regulations, and rules vary between countries and regions, and each country’s relevant laws and medical guidelines must be reviewed to confirm our findings. Finally, we should consider how these competencies fit the context in which they are embedded. The content of each competency must be further researched and refined in actual nursing practice.

## Conclusions

In this review, we analyzed the existing literature and constructed a framework of six core competencies aligned with the definition of nursing competencies, identifying the key role of nurses in the safe medication management of biologics. This can provide information and decision-making support to patients and enhance their compliance and therapeutic expectations through healthcare management and innovative nursing practices. However, more research is needed to delve deeper into the role of nurses in ensuring the safe and effective delivery of drug therapy. Furthermore, the nursing competency framework should be updated regularly to adapt to the evolution of healthcare. Future research should focus on nurses’ ability to collaborate across disciplines, the use of information technology to improve service quality, and the related role in managing biosimilars. Comparative international studies of nursing competency frameworks are also vital. Through these efforts, the competencies of nurses in biologics therapy can be fully utilized, providing direction for researchers and promoting the optimization of nursing practice and services.

## Supporting Information

S1 Table PRISMA-ScR checklist. (PDF)

S2 Table Search strategies. (DOC)

S3 Table Characteristics of the included literature. (DOCX)
