# Supplementary material for: Competencies of nurses to participate in safe medication management practices for biologics: A scoping review
Source: PLoS One. 2025 Jan 27;20(1):e0317750. doi: 10.1371/journal.pone.0317750 (PMC11771892; doi:10.1371/journal.pone.0317750)
Supplement: S2 Table — (DOC) [file pone.0317750.s002.doc]

**Appendix 2: Search strategies**

| **Online database** | **Search strategies** |
| --- | --- |
| PubMed | #1  "Nurses"[MeSH Terms] OR "Nursing Staff"[MeSH Terms] OR "Nurse Practitioners"[MeSH Terms] OR "Nurse Specialists"[MeSH Terms] OR "Nurse Clinicians"[MeSH Terms] OR "Primary Care Nursing"[MeSH Terms] OR "Nurse Administrators"[MeSH Terms] OR "Advanced Practice Nursing"[MeSH Terms] OR ("nurse s"[All Fields] OR "Nurses"[MeSH Terms] OR "Nurses"[All Fields] OR "nurse"[All Fields] OR "nurses s"[All Fields]) OR ("Nurses"[MeSH Terms] OR "Nurses"[All Fields] OR ("personnel"[All Fields] AND "nursing"[All Fields]) OR "personnel nursing"[All Fields]) OR ("Nursing Staff"[MeSH Terms] OR ("nursing"[All Fields] AND "staff"[All Fields]) OR "Nursing Staff"[All Fields] OR ("nursing"[All Fields] AND "personnel"[All Fields]) OR "nursing personnel"[All Fields] OR "Nurses"[MeSH Terms] OR "Nurses"[All Fields]) OR ("Nurses"[MeSH Terms] OR "Nurses"[All Fields] OR ("registered"[All Fields] AND "Nurses"[All Fields]) OR "registered nurses"[All Fields]) OR ("Nurses"[MeSH Terms] OR "Nurses"[All Fields] OR ("nurse"[All Fields] AND "registered"[All Fields]) OR "nurse registered"[All Fields]) OR ("Nurses"[MeSH Terms] OR "Nurses"[All Fields] OR ("Nurses"[All Fields] AND "registered"[All Fields]) OR "nurses registered"[All Fields]) OR ("Nurses"[MeSH Terms] OR "Nurses"[All Fields] OR ("registered"[All Fields] AND "nurse"[All Fields]) OR "registered nurse"[All Fields])  #2  "Nurse's Role"[MeSH Terms] OR "Clinical Competence"[MeSH Terms] OR "Professional Competence"[MeSH Terms] OR "Knowledge"[MeSH Terms] OR "Scope of Practice"[MeSH Terms] OR "role function"[Title/Abstract] OR "action fields"[Title/Abstract] OR "Scope of Practice"[Title/Abstract] OR "task domain"[Title/Abstract]  #3  "biological products"[MeSH Terms] OR ("biological"[All Fields] AND "products"[All Fields]) OR "biological products"[All Fields] OR "biologic"[All Fields] OR "biologicals"[All Fields] OR "biological factors"[MeSH Terms] OR ("biological"[All Fields] AND "factors"[All Fields]) OR "biological factors"[All Fields] OR "biologics"[All Fields] OR "biologically"[All Fields] OR "biology"[MeSH Terms] OR "biology"[All Fields] OR "biological"[All Fields] OR (("biological products"[MeSH Terms] OR ("biological"[All Fields] AND "products"[All Fields]) OR "biological products"[All Fields] OR "biologic"[All Fields] OR "biologicals"[All Fields] OR "biological factors"[MeSH Terms] OR ("biological"[All Fields] AND "factors"[All Fields]) OR "biological factors"[All Fields] OR "biologics"[All Fields] OR "biologically"[All Fields] OR "biology"[MeSH Terms] OR "biology"[All Fields] OR "biological"[All Fields]) AND ("agent"[All Fields] OR "agents"[All Fields])) OR ("biological products"[MeSH Terms] OR ("biological"[All Fields] AND "products"[All Fields]) OR "biological products"[All Fields] OR "biologic"[All Fields] OR "biologicals"[All Fields] OR "biological factors"[MeSH Terms] OR ("biological"[All Fields] AND "factors"[All Fields]) OR "biological factors"[All Fields] OR "biologics"[All Fields] OR "biologically"[All Fields] OR "biology"[MeSH Terms] OR "biology"[All Fields] OR "biological"[All Fields]) OR "biosimilar pharmace  #4  #1 AND #2 AND # 3 |
| CINAHL | #1  MH "Nursing Staff, Hospital") OR (MH "Nurses") OR (MH "Nurse Practitioners") OR (MH "Clinical Nurse Specialists") OR ( (MH "Primary Care Nurse Practitioners") OR (MH "Primary Nursing") OR (MH "Primary Health Care") ) OR (MH "Nurse Administrators") OR (MH "Advanced Practice Registered Nursing") OR nurse OR Personnel, Nursing OR Nursing Personnel OR Registered Nurses OR Nurse, Registered OR Nurses, Registered  #2  ( (MH "Scope of Practice") OR (MH "Scope of Nursing Practice") ) OR ( (MH "Knowledge") OR (MH "Nursing Knowledge") ) OR (MH "Professional Competence") OR (MH "Clinical Competence") OR ( (MH "Nursing Role") OR (MH "Nurses by Role") ) OR TI action fields OR TI role function OR TI task domain  #3  (MH "Biological Products") OR (MH "Biosimilar Pharmaceuticals") OR biologics OR biological agents OR biologicals  #4  #1 AND #2 AND # 3 |
| Embase | #1  'nurse'/exp OR 'nursing staff'/exp OR 'nurse practitioner'/exp OR 'nurse specialist'/exp OR 'clinical nurse specialist'/exp OR 'primary health care'/exp OR 'nurse administrator'/exp OR 'advanced practice nursing'/exp  #2  'clinical competence'/exp OR 'professional competence'/exp OR 'knowledge'/exp OR 'scope of practice'/exp OR 'role functioning'/exp OR 'nurse role':ab,ti OR 'action fields':ab,ti OR 'task domain':ab,ti  #3  'biosimilar agent'/exp OR biologics:ab,ti OR biologicals:ab,ti OR 'biological agents':ab,ti  #4  #1 AND #2 AND #3 |
| Scopus | ((TITLE-ABS-KEY(nurse) OR TITLE-ABS-KEY(nursing staff) OR TITLE-ABS-KEY(nurse practitioners) OR TITLE-ABS-KEY(nurse specialists) OR TITLE-ABS-KEY(nurse clinicians) OR TITLE-ABS-KEY(primary care nursing) OR TITLE-ABS-KEY(nurse administrators) OR TITLE-ABS-KEY(advanced practice nursing) OR TITLE-ABS-KEY(nursing personnel) OR TITLE-ABS-KEY(registered nurses))) AND ((TITLE-ABS-KEY(competence) OR TITLE-ABS-KEY(scope of practice) OR TITLE-ABS-KEY(knowledge) OR TITLE-ABS-KEY(role functioning) OR TITLE-ABS-KEY(nurse role) OR TITLE-ABS-KEY(action fields) OR TITLE-ABS-KEY(task domain)))AND ((TITLE-ABS-KEY(biologics) OR TITLE-ABS-KEY(biologicals) OR TITLE-ABS-KEY(biological agents) OR TITLE-ABS-KEY(biosimilar))) |
| Web of Science | #1  TS: ("nurses") OR TS: ("nursing staff") OR TS: ("nurse practitioners") OR TS: ("nurse specialists") OR TS: ("nurse clinicians") OR TS: ("primary care nursing") OR TS: ("nurse administrators") OR TS: ("advanced practice nursing")  #2  TS: ("nurse's Role") OR TS: ("clinical competence") OR TS: ("professional competence") OR TS: ("knowledge") OR TS: ("scope of practice") OR TS: ("role function") OR TS: ("action fields") OR TS: ("task domain")  #3  TI: ("biologics") OR TI: ("biologicals") OR TI: ("biological agents") OR TI: ("biosimilar")  #4  #1 AND #2 AND #3 |
