# Supplementary material for: Competencies of nurses to participate in safe medication management practices for biologics: A scoping review
Source: PLoS One. 2025 Jan 27;20(1):e0317750. doi: 10.1371/journal.pone.0317750 (PMC11771892; doi:10.1371/journal.pone.0317750)
Supplement: S3 Table — (DOCX) [file pone.0317750.s003.docx]

**S3 Table Competencies of nurses to participate in safe medication management practices for biologics.(n=24)**

| **Author,Year,**  **Country** | **Objective** | **Design** | **nurses’ competencies in NSSP** | | | | **Safe medication management practices relevant competencies** |
| --- | --- | --- | --- | --- | --- | --- | --- |
|  |  |  | **A** | **B** | **C** | **D** |  |
| Trettin et al[1].  2021,  Denmark | To investigate psoriasis patients’ and healthcare professionals’ experiences and  perspectives of follow-up consultations | Qualitative study  (focus group  discussions,  observation, and  semi-structured  interviews | + | - | + | + | Assessment (signs and symptoms, signs of infection);  Safety management (recognition and management of infusion reactions, management of side effects);  Monitoring (monitoring of medication, monitoring of vital signs);  Health education (self-injection);  Professional knowledge (storage of medication, dosage and frequency of medication, common side effects);  Provision of premedication; Prudence |
| Scalone et al[2].  2018,  Italy | To estimate preferences in relevant treatment characteristics evaluated by different  groups involved in the management of patients with rheumatic diseases | Quantitative study: a discrete choice experiment | + | + | + | + | Specialized knowledge (knowledge of disease, therapeutic interventions, working knowledge of diagnostic procedures, treatment side effects, legal knowledge);  Safety management (pre-treatment information assessment and screening, infusion management);  Psychological support;  Health education (nursing and side effects);  Pretreatment documentation;  Communication (development of linkage to nursing roles); Health education (self-administration training, side effect education);  Multidisciplinary review and management (virtual biologics clinic);  Evidence-based practice;  Medication safety monitoring (drug side effects, potential complications, clinical and biochemical reactions) |
| Taylor et al[3].  2016,  UK | Develop a specialist nurseled biologics service to improve patient care. | Qualitative research | + | + | + | + | Screening (infection risk, medical history);  Assessment for optimal nutritional status;  Information technology;  Co-ordination of referrals;  Counselling and education;  Liaison with infusion day unit;  Injection technology and support;  Communication and liaison;  Blood monitoring;  Coordination of regular outpatient follow-up;  Audit and clinical management;  Multidisciplinary collaboration (team review, virtual clinic) |
| Melis et al[4].  2023,  Italy | Investigating unmet needs and identifying the necessary interventions for patients affected by rheumatic and musculoskeletal  diseases (RMDs) may help significantly to ensure the continuity and quality of the chronic care pathway | Review | + | + | + | - | Education(self-administration,multidisciplinary education intervention,symptoms,self-management,strategies,treatme-nt options,side effects of medications);  Assessment(psychiatric symptoms);  Telenursing;  Patient-centred care(treatment monitoring);  Data collection;  Psychological support;  Communication (shared decision-making, referrals); Medication management for special populations;  Telecare (DVDs and teleconferencing) |
| Bokemeyer et al[5].  2024,  Germany | To evaluate patient-related outcomes and economic consequences associated  with integrating IBD nurses into usual care | Quantitative research  (RCT) | - | + | + | + | Bridging the communication gap;  Providing consultations (psychosocial problems, management of drug therapy);  Information assessed;  Psychosocial support;  Coordination skills;  Record electronic medical |
| Greveson et al[6].  2013,  UK | Coordinating and managing a biologics  service requires highly skilled specialist nursing knowledge and  awareness of the support, assessment, administration and monitoring  required in supporting the patient through this pathway. | Descriptive study | + | + | + | + | Treatment funding (aid the funding process, manage treatment pathway);  Education(pre-treatment counselling, self-management, shared decision-making, risk of medication in special populations, application of evidence-based tools);  Screening investigationsare performed;  Medication management;  Monitoring and assessment (adverse reactions or contraindications,response to treatment);  Data collection and audit;  Multi-disciplinary team (virtual biologics clinics);  Clinical Specialized knowledge (manage infusion reactions,well-documented,choice and mode of therapy, use decision aids);  Ambulatory infusion services;  Communication and collaboration;  Network support and information sharing;  Professional nursing knowledge;  Pretreatment records |
| Larsson et al[7].  2015,  Sweden | To compare the costs  of rheumatology care between a nurse-led rheumatology clinic (NLC), based on person-centred care (PCC), versus  a rheumatologist-led clinic (RLC), in monitoring of patients with CIA undergoing biological therapy. | Quantitative research  (RCT) | + | + | + | - | Medication monitoring (pharmacological therapy, adherence, side effects and blood tests);  Multidisciplinary team cooperation;  Professional knowledge;  Health education (providing information about the disease); Communication (shared decision-making);  Patient-centred care |
| Larsson et al[8].  2014,  Sweden | To compare and evaluate the treatment outcomes of a nurse-led  rheumatology clinic and a rheumatologist-led clinic in patients with low disease  activity or in remission who are undergoing biological therapy | Quantitative research  (RCT) | + | + | + | + | Education(self-administration);  Evidence-based care(nursing knowledge and competence);  Nurse-patient collaboration(information sharing);  Medication management (safe administration of medication, assessment of medication efficacy, disease activity); Communication and cooperation (shared decision-making, psychological guidance);  Ambulatory infusion services; Professional knowledge; Person-centred care;  Meeting individual needs (lifestyle and psychosocial aspects) |
| Thomaidou et al[9].  2019,  Israel | To review the  different aspects of injection site reactions, including their epidemiology and pathogenesis, and provides practical guidance to diagnose and treat such reactions. | Retrospective study | + | + | + | - | Specialized knowledge(injection site reactions,type of reaction,properties of the injected drug);  Ability to use drugs safely(injection techniques,injection site,Post-injection treatment);  Education and counselling;  Assessed(ability to inject);  Drug management(Drug storage);  Education and counselling;  Assessed(ability to inject) |
| Bittner et al[10].  2018,  Switzerland | To discuss the differences between  subcutaneous and intravenous dosing from both health-economic and scientific perspectives. | Review | + | - | + | + | Specialized knowledge(injection site,standard administration route,Dosing frequency,Injection volume, reaction symptoms, injection site;  Education(self administration,concomitant medications);  Assessed(Life Quality Index);  Telecare;Communication(understanding patient's injection preferences);  Mastery of infusion equipment technology;  Timely management of infusion reactions |
| Rinaldi et al[11].  2021,  USA | To better  understand current practices in biologic initiation and self-injection training, identify areas for improvement, and characterize innovation opportunities for both patients and  HCPs. | Mixed methods | + | + | + | + | Specialized knowledge (technical steps, injection sites, adverse reactions, dosing frequency, injection preparation information, drug safety information/side effects);  Medication monitoring (emotions/injection-related anxiety); Mastery of communication skills;  Education (self-administration);  Medication monitoring (emotions/injection-related anxiety); Mastery of communication skills; Education(self-administration,provision of training resources,development of multi-form teaching); Psychological support;  Healthcare collaboration |
| Vizgirda et al[12].  2017,  USA | To review the properties of biosimilars; their regulation and approval process;  the ways in which their quality, safety, and efficacy  are evaluated; their postmarketing safety monitoring; and their significance to oncology nurses and  oncology nursing | Review | + | + | + | - | Education(biosimilars'development,safety,and efficacy,self-monitoring,safe use skills);  Collaborate with pharmacists;Evaluating and monitoring(pharmacovigilance,adverse events);  Specialized knowledge(administration, handling, and/or storage,generic mechanism of action function and differences);  Drug switching;  Monitoring( recognise and record adverse events); Pharmacovigilance (detection, assessment, prevention); Building trust with patients and families |
| O’Malley et al[13].  2017,  USA | To determine the biologics in clinical nurse specialist practice | Descriptive study | + | - | + | + | Specialized knowledge;  Assessment(Screening, reviewing risks and benefits with treatment options,assessment of risks);  Educational support;  Monitoring(allergic reactions and/or sensitivities,current, recurrent or latent infections);  Pre-drug switching assessments |
| Wolf et al[14].  2018,  USA | To assess, using real-world claims data, whether home health nurse assistance  had an effect on patients’ adherence to CZP and to measure its impact on health care use  and costs | Retrospective cohort study | + | + | + | + | Injecting skills;  Self-injection teaching;  Regular follow-up;  Assessed and observed (comorbid condition, medication history et al);  Psychological support (identification of abnormal psychological states) |
| Palmer et al[15].  2010,  UK | To discuss the evolution of the IMID/biologic specialist nurse role and how IMID  services started with goodwill from the rheumatology nurse specialists to develop  into a main component of the holistic approach to care. | Descriptive study | + | + | + | + | Multidisciplinary team cooperation;  Assessed(contraindications,disease activity);  Identify and management(disease activity symptoms/signs and side-effects/adverse reactions);  Education and Train patients (self-administration);  Specialized knowledge;  Collaborate with others;  Communicate (Manage a telephone advice helpline); Manage a day ward and record relevant data;  Telecare |
| Beauvais et al[16].  2022,  France | To evaluate the effect of a nurse-led patient  education on safety skills of patients with inflammatory  arthritis treated with biologic disease-modifying  antirheumatic drugs (bDMARDs). | Quantitative research  (RCT) | + | + | + | - | Safety training;  Educational interventions (safety training, self-care skills); Assessment (potential safety threats, Patients' problem-solving abilities);  Communication skills;  Medication knowledge;  Data interpretation and problem solving |
| Gall et al[17].  2022,  Germany | To assess patients’ satisfaction after  being educated about switching of bsDMARDs by rheumatologists compared to nurse specialists, and to explore the impact of multiple switches on patient  satisfaction. | Quantitative research  (Semi-structured interviews) | + | - | + | + | Specialized knowledge;  Recognition and management of adverse events;  Provision of information counselling(the mode of action,side-effects of drugs);  Clinical Specialized knowledge(based on the principles of evidence-based practice);  Education(self-management skills, drug switching);  Use of educational aids |
| Aldredge et al[18].  2016,  USA | To optimize clinical efficacy  outcomes and consistently manage moderate-to-severe  psoriasis and its comorbidities over the patient’s life course. | Review | + | + | + | - | Specialized knowledge (disease knowledge, drug knowledge);  Psychological support;  Communication skills;  Education (self-management, identification and management of side effects);  Assessment (treatment satisfaction and adherence, infections, psychological stress);  Teamwork;  Nursing practice skills;  Psychosocial support(patients with a fear of needles);  Telecare (e-communication, application of information technology) |
| Sehdev et al[19].  2019,  Canada | To assist  Canadian nurses in gaining a greater understanding of biosimilars within the oncology treatment landscape and to alleviate certain concerns regarding biosimilar agents. | Editorial | + | + | + | + | Specialized knowledge (knowledge of biosimilars); Communication (support for shared decision-making); Education and support (medication switching, side effect recognition, patient reassurance);  Monitoring of adverse events;  Medication switching management;  Multidisciplinary collaboration (effective communication); Familiarity with and adherence to regional policies;  Telecare (teleconferencing);  Pharmacovigilance reporting |
| Corominas et al[20].  2013,  Spain | To confirm the safety profile of biological therapies in routine clinical practice, after the  administration of intravenous drugs and 24 h post-administration. | Quantitative study  (observational cohort study) | + | + | + | + | Specialized knowledge; assessment screening (signs of infection);  Ongoing monitoring (signs of infection, drug efficacy, body weight);  Health education (self-injection instructions);  Safe handling (administration of medication, monitoring of vital signs to identify and manage adverse events); Psychological support (patient concerns about treatment); Medication management (storage, preparation);  Effective liaison with multidisciplinary team |
| Baxter et al[21].  2023,  USA | To ensure patient safety and maximizing the number of patients  who benefit from a full treatment course of pegloticase. | Qualitative study | - | + | + | + | Screening(contraindications);  Monitoring(monitoring of relevant indicators, medication adherence);  Safety management (emergency management of infusion reactions, premedication);  Medication management (storage, dispensing);  Provision of counselling and education;  Risk management |
| Guarini et al[22].  2016,  Italy | To assess the presence of IBD nurse in centers where patients were receiving biologics | Quantitative study  (Cross-sectional survey) | + | + | + | - | Screening (premedication screening for relevant indicators); Monitoring (infusion process);  Health education;  Multidisciplinary collaboration;  Training(patient self-injection);  Telecare (online follow-up);  Programme control |
| Waller et al[23].  2020,  Germany, | To help prepare nurses to respond to potential questions from patients on biosimilars. | Qualitative study | + | + | + | + | Specialized knowledge;  Patient education;  Assessment (clinical assessment, pharmacological assessment);  Counselling (biosimilar related information);  Therapeutic monitoring (side effects);  Development of risk management plans (identification and management of adverse events);  Communication; pharmacovigilance |
| Friganovi´c et al[24].  2022,  Switzerland. | To assess the knowledge and attitudes of nurses towards biosimilar drugs in different countries. | Quantitative study  (Cross-sectional survey) | + | + | + | + | Specialized knowledge (drug injection sites, dose, frequency, adverse effects of combinations);  Monitoring (risk of TB reactivation, liver function, body weight, adverse events);  Safety of care (management of adverse effects of infusions); Assessment (response to therapy and efficacy); Communication (understanding patient preferences and values);  Regular follow-up;  Education (use of internet resources to provide education, management of side-effects, risks of infection);  Special populations Management |

**References**

1. Trettin B，Feldman S，Andersen F，et al. Improving management of psoriasis patients receiving biological treatment: A qualitative approach [J]. Nurs Open, 2021,8(3): 1283-1291.PMID:33385301

2. Scalone L，Sarzi-Puttini P，Sinigaglia L，et al. Patients', physicians', nurses', and pharmacists' preferences on the characteristics of biologic agents used in the treatment of rheumatic diseases [J]. Patient Prefer Adherence, 2018,12: 2153-2168.PMID:30410311

3. Taylor N S，Bettey M，Wright J，et al. The impact of an inflammatory bowel disease nurse-led biologics service [J]. Frontline Gastroenterol, 2016,7(4): 283-288.PMID:28839869

4. Melis M R，El Aoufy K，Bambi S，et al. Nursing interventions for patients with rheumatic and musculoskeletal diseases on biological therapies: a systematic literature review [J]. Clin Rheumatol, 2023,42(6): 1521-1535.PMID:36801986

5. Bokemeyer B，Plachta-Danielzik S，Steiner I M，et al. Inflammatory bowel disease (IBD) patients with impaired quality of life on biologic therapy benefit from the support of an IBD nurse specialist: Results of a randomised controlled trial in Germany (IBD(BIO-ASSIST) study) [J]. Aliment Pharmacol Ther, 2024,59(9): 1082-1095.PMID:38429885

6. Greveson K，Woodward S. Exploring the role of the inflammatory bowel disease nurse specialist [J]. Br J Nurs, 2013,22(16): 952-954, 956-958.PMID:24037398

7. Larsson I，Fridlund B，Arvidsson B，et al. A nurse-led rheumatology clinic versus rheumatologist-led clinic in monitoring of patients with chronic inflammatory arthritis undergoing biological therapy: a cost comparison study in a randomised controlled trial [J]. BMC Musculoskelet Disord, 2015,16: 354.PMID:26573936

8. Larsson I，Fridlund B，Arvidsson B，et al. Randomized controlled trial of a nurse-led rheumatology clinic for monitoring biological therapy [J]. J Adv Nurs, 2014,70(1): 164-175.PMID:23772698

9. Thomaidou E，Ramot Y. Injection site reactions with the use of biological agents [J]. Dermatol Ther, 2019,32(2): e12817.PMID:30637967

10. Bittner B，Richter W，Schmidt J. Subcutaneous Administration of Biotherapeutics: An Overview of Current Challenges and Opportunities [J]. BioDrugs, 2018,32(5): 425-440.PMID:30043229

11. Rinaldi A，Katsaros D，Hawthorne J，et al. The current paradigm for biologic initiation: a mixed-methods exploration of practices, unmet needs, and innovation opportunities in self-injection training [J]. Expert Opin Drug Deliv, 2021,18(8): 1151-1168.PMID:33896303

12. Vizgirda V，Jacobs I. Biosimilars: Considerations for Oncology Nurses [J]. Clin J Oncol Nurs, 2017,21(2): E54-e60.PMID:28315542

13. O'Malley P A. Large Molecule Pharmacotherapy: Biologics in Clinical Nurse Specialist Practice [J]. Clin Nurse Spec, 2017,31(3): 134-137.PMID:28383330

14. Wolf D C，Jaganathan S，Burudpakdee C，et al. Adherence rates and health care costs in Crohn's disease patients receiving certolizumab pegol with and without home health nurse assistance: results from a retrospective analysis of patient claims and home health nurse data [J]. Patient Prefer Adherence, 2018,12: 869-878.PMID:29872272

15. Palmer D，El Miedany Y. Biological nurse specialist: goodwill to good practice [J]. Br J Nurs, 2010,19(8): 477-480.PMID:20505612

16. Beauvais C，Fayet F，Rousseau A，et al. Efficacy of a nurse-led patient education intervention in promoting safety skills of patients with inflammatory arthritis treated with biologics: a multicentre randomised clinical trial [J]. RMD Open, 2022,8(1).PMID:35296528

17. Gall S，Kiltz U，Kobylinski T，et al. Patient knowledge about biosimilars and satisfaction with the education provided by rheumatologists or nurse specialists in a biosimilar multiswitch scenario - The perception study [J]. Semin Arthritis Rheum, 2022,57: 152119.PMID:36341778

18. Aldredge L M，Young M S. Providing Guidance for Patients With Moderate-to-Severe Psoriasis Who Are Candidates for Biologic Therapy: Role of the Nurse Practitioner and Physician Assistant [J]. J Dermatol Nurses Assoc, 2016,8(1): 14-26.PMID:27004085

19. Sehdev S，Perry K，Gesy K. Biosimilars in oncology in Canada and the role of nurses [J]. Canadian Oncology Nursing Journal/Revue canadienne de soins infirmiers en oncologie, 2019,29: 1-11

20. Corominas H，Sánchez-Eslava L，García G，et al. Safety profile of biological intravenous therapy in a rheumatoid arthritis patients cohort. Clinical nursing monitoring (Sebiol study) [J]. Reumatol Clin, 2013,9(2): 80-84.PMID:2309928521. Baxter B，Sanders S，Patel S A，et al. Pegloticase in Uncontrolled Gout: The Infusion Nurse Perspective [J]. J Infus Nurs, 2023,46(4): 223-231.PMID:37406337

21. Baxter B，Sanders S，Patel S A，et al. Pegloticase in Uncontrolled Gout: The Infusion Nurse Perspective [J]. J Infus Nurs, 2023,46(4): 223-231.PMID:37406337

22. Guarini A，Marinis F，Kohn A，et al. Inflammatory bowel disease nurse specialists for patients on biological therapies: a nationwide Italian survey [J]. Ann Gastroenterol, 2016,29(4): 492-496.PMID:27708516

23. Waller C F，Friganović A. Biosimilars in oncology: key role of nurses in patient education [J]. Future Oncol, 2020,16(25): 1931-1939.PMID:32618476

24. Friganović A，Mędrzycka-Dąbrowska W，Krupa S，et al. Nurses' Knowledge and Attitudes towards Biosimilar Medicines as Part of Evidence-Based Nursing Practice-International Pilot Study within the Project Biosimilars Nurses Guide Version 2.0 [J]. Int J Environ Res Public Health, 2022,19(16).PMID:36011946
